# Lysophosphatidic Acid–Induced EGFR Transactivation Promotes Gastric Cancer Cell DNA Replication by Stabilizing Geminin in the S Phase

**DOI:** 10.3389/fphar.2021.706240

**Published:** 2021-09-29

**Authors:** Haile Zhao, Gezi Gezi, Xiaoxia Tian, Peijun Jia, Morigen Morigen, Lifei Fan

**Affiliations:** State Key Laboratory of Reproductive Regulation & Breeding of Grassland Livestock, School of Life Sciences, Inner Mongolia University, Hohhot, China

**Keywords:** LPA, EGFR, transactivation, DNA replication, geminin

## Abstract

Geminin, an inhibitor of the DNA replication licensing factor, chromatin licensing and DNA replication factor (Cdt) 1, is essential for the maintenance of genomic integrity. As a multifunctional protein, geminin is also involved in tumor progression, but the molecular details are largely unknown. Here, we found that lysophosphatidic acid (LPA)–induced upregulation of geminin was specific to gastric cancer cells. LPA acted *via* LPA receptor (LPAR) 3 and matrix metalloproteinases (MMPs) signaling to transactivate epidermal growth factor receptor (EGFR) (Y1173) and thereby stabilize geminin expression level during the S phase. LPA also induced the expression of deubiquitinating protein (DUB) 3, which prevented geminin degradation. These results reveal a novel mechanism underlying gastric cancer progression that involves the regulation of geminin stability by LPA-induced EGFR transactivation and provide potential targets for the signaling pathway and tumor cell–specific inhibitors.

## Introduction

Precise control of DNA replication is critical for maintaining genomic integrity. To this end, both eukaryotic and prokaryotic cells have evolved various mechanisms to ensure that nuclear DNA is completely replicated at the right time, right place, and only once per cell division (Machida et al., 2005; Zhou et al., 2020). Initiation of DNA replication is strictly regulated in eukaryotes, as reinitiation at any starting origin leads to cell death or genomic rearrangement, which causes serious physiologic consequences, including cancer. The regulatory mechanism involves suppression of chromatin licensing and DNA replication factor (Cdt)1 function by geminin, proteasome-mediated Cdt1 degradation in the S phase, and cyclin-dependent kinase (CDK)–mediated inhibition of transcription initiation (Miotto and Struhl, 2010; Wong et al, 2010; Diffley, 2011; Ballabeni et al, 2013).

Geminin directly binds to Cdt1 and inhibits pre-replicative complex formation to prevent aneuploidy and re-replication (McGarry and Kirschner, 1998; Wohlschlegel et al., 2000). The precise control of geminin levels throughout the cell cycle mainly depends on ubiquitin-mediated proteasomal degradation. Geminin levels fluctuate throughout the cell cycle and the protein is degraded in late mitosis/G1 phase by the anaphase-promoting complex/cyclosome (APC/C) ubiquitin ligase complex to facilitate origin licensing (Wohlschlegel et al., 2000). In contrast, geminin accumulates in early S, G2, and early M phases as a result of APC/C ubiquitin ligase complex inhibition, ensuring that pre-replicative complex formation is blocked (Ballabeni et al., 2004). A recent study reported that in the late S phase, the microRNA miR-571 reduced geminin protein level via the Cdk2–c-Myc–miR-571 axis independent of the APC/C inducing DNA replication efficiency and S-phase cell-cycle progression (Zhang et al., 2019). In the early M phase, Aurora-A phosphorylates geminin at Thr25 to prevent its degradation by APC/C during mitosis (Tsunematsu et al., 2013). Deubiquitinating protein (DUB)3 and ubiquitin-specific peptidase (USP) 7 also increase geminin levels by preventing its ubiquitination (Hernández-Pérez et al., 2017).

Geminin is a multifunctional protein that plays important roles in DNA replication and transcriptional/epigenetic regulation and is frequently deregulated in human cancers. The upregulation of geminin in various malignancies was shown to be correlated with tumor cell proliferation, invasion, and metastasis (Gonzalez et al., 2004; Petropoulou et al., 2008; Emmett and O’Shea, 2012; Rajan et al., 2013; Sato et al., 2013; Hills and Diffley, 2014; Yagi et al., 2014; Zhang et al., 2017; Ryan et al, 2019; Alaeddini and Etemad-Moghadam, 2020; Lewis and Stracker, 2021). Tumor cells depend on geminin to prevent excess DNA replication from triggering DNA damage–induced apoptosis. Knockdown of geminin resulted in DNA re-replication and apoptosis in malignant cancer cells, whereas normal and immortalized cells were unaffected (Zhu and Depamphilis, 2009). Aberrant geminin expression has been linked to DNA replication damage, aneuploidy, and genomic instability, all of which are associated with a precancerous state and malignant transformation (Petropoulou et al., 2008; Champeris Tsaniras et al., 2018). Thus, geminin is considered as an oncogene, although its mechanism of action in tumorigenesis is not fully understood.

The tumor microenvironment (TME) includes various cell types and extracellular components. Communication between tumor cells and the TME involves cell–cell and cell-extracellular matrix (ECM) adhesion, as well as cellular responses to soluble molecules (Wu and Dai, 2016). The TME is implicated in tumor initiation, progression, and metastasis. Soluble extracellular components such as lysophospholipase D autotaxin (ATX) and its product lysophosphatidic acid (LPA) are key factors in cancer progression. *In vitro* and *in vivo* studies have shown that increased ATX/LPA signaling contributes to cancer initiation and progression (Mills and Moolenaar, 2003; Leblanc and Peyruchaud, 2014; Lee et al., 2015). LPA stimulates cell proliferation, migration, and survival through activation of G protein–coupled receptors (GPCRs). Both LPA receptor (LPAR) family members and ATX are aberrantly expressed in various malignancies, including breast, ovarian, and prostate cancers, hepatocellular carcinoma multiforme, and melanoma (Lee et al., 2015). The context of LPA biology is complex as it involves not only several distinct GPCRs, but also cross-talk with receptor tyrosine kinase signaling via matrix metalloproteinase (MMP) activation (Gschwind et al., 2002; Köse, 2017). Epidermal growth factor receptor (EGFR) transactivation by GPCRs was shown to induce mitogen-activated protein kinase (MAPK) signaling and gene expression, stimulate DNA synthesis, and regulate cell-cycle progression (Daub et al., 1996; Kalmes et al., 2000; Alsahafi et al, 2020); and transactivation of EGFR by LPA and sphingosine-1-phosphate has been linked to the pathophysiology of human cancer (Gschwind et al., 2002; Deng et al, 2004; Sukocheva et al., 2006; Tveteraas et al, 2016), although the mechanisms are yet to be elucidated clearly.

LPA is significantly elevated in the plasma and ascites of gastric cancer patients with peritoneal carcinomatosis compared to healthy individuals (Zeng et al., 2017). Submucosal connective tissue–type mast cells are a source of LPA in the gastrointestinal tract (Mori et al., 2007). LPA stimulates gastric cancer cell migration and invasion *via* various effectors (Murray et al., 2008; Ren et al., 2019; Shida et al., 2008), as well as cell proliferation by upregulating sphingosine kinase (SPHK) 1 transcription (Ramachandran et al., 2010). However, it is unclear whether LPA affects DNA replication in gastric cancer.

In this study, we investigated the role and mechanism of action of geminin and LPA in gastric cancer. Our results indicated that geminin functions as a regulator of gastric cancer progression. Depletion of geminin induced DNA re-replication in gastric cancer cells. Meanwhile, LPA induced the upregulation of geminin protein and induced EGFR transactivation via an MMP-dependent pathway, which was partly responsible for stabilizing geminin in the early S phase and promoting DNA replication. These data indicate that the cross-talk between LPA and EGFR signaling pathway regulates DNA replication in gastric cancer cells by controlling geminin levels.

## Materials and Methods

### Cell Culture

Gastric cancer cells (MKN45 and BGC-803) were maintained in RPMI 1,640 medium (Gibco) supplemented with 10% fetal bovine serum (BI) and 1% (vol/vol) penicillin/streptomycin/L-Glutamin (Gibco). Human gastric mucosal epithelial cells (GES-1) were maintained in DMEM (High glucose, Gibco) supplemented with 10% (vol/vol) fetal bovine serum (BI) and 1% (vol/vol) penicillin/streptomycin/L-Glutamin (Gibco). All cells were cultured at 37°C and 5% CO_2_ in a humidified atmosphere (Thermo Fisher Scientific, MA, United States). To synchronize cells in the G1/S-phase, the cells were cultured in serum-free medium for 24 h.

### Reagents and Inhibitors

Reagents and inhibitors were purchased from the following suppliers: LPA (SIGMA), DMSO (SIGMA), BB94 (SIGMA), BSA (SIGMA), Ki16425 (SELLECK), AG1478 (SELLECK), LY294002 (SELLECK), and Rapamycin (SELLECK).

### RNA Interference

To knockdown endogenous geminin expression, the cells were transfected with 50 nM siRNA that targets human geminin using Lipofectamine 2000 (Thermo Fisher Scientific, MA, United States ). Three oligos of siGEM were mixed in equal proportions. Short interfering oligoribonucleotide for luciferase (non-targeting siRNA, siGL/siNC/sitrl) was used as a control. Both of them were used as previously described (Zhu et al., 2004; Zhu and Depamphilis, 2009) and obtained from Sangon Biotech (Shanghai, China) and their sequences are listed in Supplementary Table 1. Knockdown efficiency of endogenous geminin expression was confirmed by Western blotting using an antibody against geminin.

To knockdown endogenous LPAR_3_ expression, the cells were transfected with 50 nM siRNA that targets human LPAR_3_ using Lipofectamine 2000. Nontargeting siRNA (sitrl) was used as a control. The custom-synthesized siRNA sequences were ordered from Sangon and their sequences are listed in Supplementary Table 1. Knockdown efficiency of endogenous LPAR_3_ expression was confirmed by quantitative real-time (qRT)-PCR.

To knockdown endogenous DUB3 expression, the cells were transfected with 50 nM siRNA that targets human DUB3 using Lipofectamine 2000. Nontargeting siRNA (siNC) was used as a control. Both of them were used as previously described (Hernández-Pérez et al., 2017). The custom-synthesized siRNA sequences were ordered from Sangon and their sequences are listed in Supplementary Table 1. Knockdown efficiency of endogenous DUB3 expression was confirmed by western blotting using an antibody against DUB3.

### Flow Cytometry Analysis of Cell Cycle

Cell-cycle progression under different conditions was evaluated by flow cytometry. After treatment, cells were harvested by digestion with 0.05% trypsin, washed twice with ice-cold 1× phosphate-buffered saline (1x PBS), and then fixed overnight at −20°C in 70% ethanol. The next day, the cells were washed with 1x PBS and incubated with 50 μg/ml propidium iodide (PI) and 50 μg/ml RNase A in 1x PBS on ice for 30 min in the dark. Flow cytometry was performed on a FACSCalibur system (BD Biosciences, San Jose, CA, United States) with CELLQuest v3.3 software (BD Biosciences), and cell-cycle distribution was analyzed with ModFit LT v3.0 software (Verity Software House, Topsham, ME, United States).

### RNA Extraction, Reverse Transcription PCR, and qRT-PCR

Total RNA was extracted from cells using TRIzol (TransGen Biotech, Beijing, China) according to the manufacturer’s instructions. TransScript One-Step gDNA Removal and cDNA Synthesis SuperMix (TransGen Biotech) was used to generate cDNA template, and qRT-PCR was performed with the TransStart Tip Green qPCR SuperMix (TransGen Biotech) on a LightCycler 480 II system (Roche, Basel, Switzerland). The mRNA expression levels of target genes were calculated relative to that of β-actin. The primers were obtained from Sangon and their sequences are listed in Supplementary Table 2.

### Western Blotting and Immunoprecipitation

Cells were lysed with lysis buffer (250 mM Tris [pH 6.8], 20 mM DL-dithiothreitol, 150 mM bromophenol blue, 10% [v/v] glycerol, and 1% sodium dodecyl sulfate [SDS]). Immunoprecipitation was performed as described in this article. Proteins in the cell lysates were separated by SDS–polyacrylamide gel electrophoresis and electrotransferred to a semi-dry membrane (Bio-Rad, Hercules, CA, United States), then incubated with antibodies and exposed to Pierce ECL Western Blotting Substrate (Thermo Fisher Scientific) for visualization of protein bands. The relative amounts of geminin and other proteins were quantified by densitometry using the ChemiDoc XRS system (Bio-Rad) and normalized in that of β-tubulin. The geminin/tubulin Western blotting signal ratios were calculated using Image Lab Version 5.2.1 (Bio-Rad) for each time point. Densitometry of three independent experiments was performed, normalized to tubulin, and expressed as a ratio of the control lane of each group. The following antibodies were used: Geminin rabbit polyclonal antibody (Proteintech), EGFR-specific rabbit polyclonal antibody (Proteintech), rabbit anti-DUB3 polyclonal antibody (Proteintech), mouse anti-β-tubulin monoclonal antibody (Transgen), anti-phosphotyrosine antibody (abcam), goat anti-rabbit IgG (Transgen), and goat anti-mouse IgG (Transgen). In Western blotting, dilution ratio for all primary antibodies was 1:1,000 and 1:25,000 for all secondary antibodies. For the immunoprecipitation assay, the antibody was used at 1:100 dilution.

### Immunofluorescence Analysis and Confocal Microscopy

Cells grown on glass coverslips in a 12-well plate were fixed in 4% paraformaldehyde (PFA) for 15 min, permeabilized with Triton X-100 for 5 min, and incubated in sodium borohydride for 10 min. They were then incubated with rabbit anti-geminin antibody (1:200) in 1x PBS containing 1% bovine serum albumin (BSA) for 1 h at room temperature (RT). Cells were washed 5 times with 1x PBS, incubated with Alexa Fluor 488–conjugated goat anti-rabbit IgG (H + L) secondary antibody (1:200; Invitrogen, Carlsbad, CA, United States ) in 1x PBS containing 1% BSA for 45 min at RT in the dark, followed by washing twice with 1x PBS. DNA was visualized by staining with 4’,6-diamidino-2-phenylindole (1:5,000 in water; Invitrogen). Cells were visualized with a laser scanning confocal microscope (LSM 710; CarlZeiss, Wetzlar, Germany).

### Cell Viability Staining

Gastric cancer cells were cultured on sterile glass coverslips in a 12-well plate, and after the transfections of siGL or siGEM, all cells were cultured for 2 days. Cells were visualized by using Live & Dead Viability/Cytotoxicity Assay Kit for Animal Cells (KeyGEN BioTECH, China). Cells were then visualized by using laser scanning confocal microscope (CarlZeiss LSM 710).

### Cell Proliferation Assay

Cells were seeded on a 96-well plate with 2,000 cells for each well and grown in 200 μl serum-free medium with 0.1% DMSO, 10 μM LPA, or 10 ng/ml EGF. The medium was changed every 2 days. 20 µl of Cell Counting Kit-8 (CCK-8) was provided to every well for 1.5 h. The plate was then read using a spectrophotometric microtiter plate reader (EPOCH) set at a dual wavelength of 450 nm. Bromodeoxyuridine (BrdU) is incorporated into newly synthesized DNA strands of actively proliferating cells (Abcam, ab126556, BrdU Cell Proliferation ELISA Kit [colorimetric]).

### Statistical Analyses

All statistical analyses were processed using GraphPad Prism v8.0 software. Quantitative analyses from three independent experiments (mean ± SD) are shown. More than two groups were analyzed for data by ANOVA test and Dunnett’s multiple comparisons test under the assumption of normality. In data with two groups, unpaired Student’s t-tests were used under the assumption of normality. In general, at least three independent biological replicates were carried out for each experiment. *p < 0.05, **p < 0.01, and ***p < 0.001 were considered statistical significance.

### URLs

The Cancer Genome Atlas (TCGA), http://cancergenome.nih.gov/. Human Protein Atlas (HPA), http://www.proteinatlas.org/.

## Results

### Geminin is Not Frequently Mutated in Human Cancers

**FIGURE 1:**
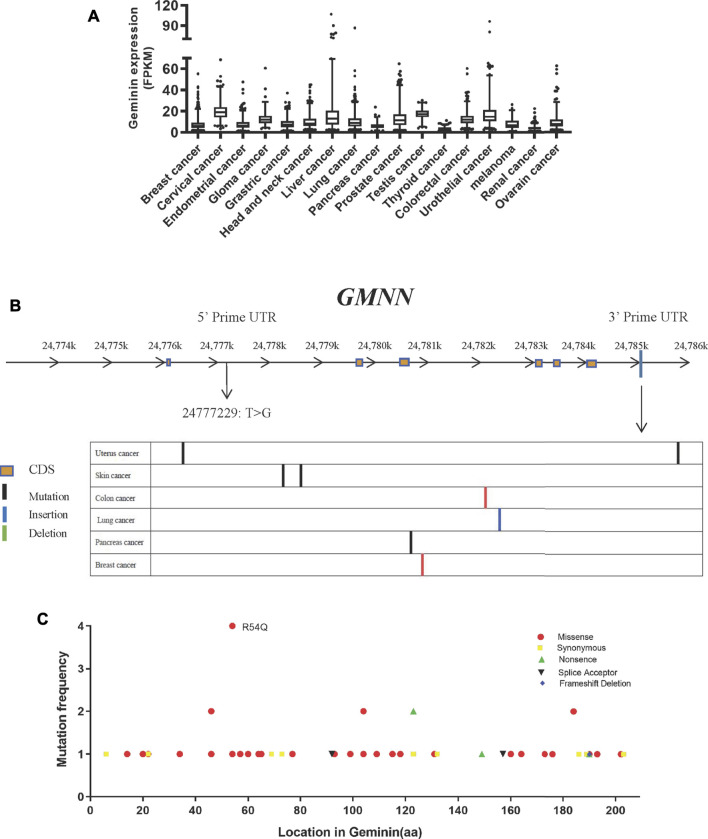
Mutation of geminin is non-predominant in human cancer. **(A)** RNA expression of geminin in 17 cancer types. **(B)** Location of mutation sites in *GMNN* gene. **(C)** Summary of geminin mutations across different tumor types from TCGA.

To explore the genetic abnormalities affecting the gene encoding geminin (*GMNN*) in human cancers comprehensively, we searched public databases including the TCGA and HPA (Uniform Resource Locators, URLs). The *GMNN* mRNA expression level was similar across 17 cancer types (Figure 1A), indicating that the gene has no cell or tissue specificity, consistent with its function as a regulatory protein that is expressed during the proliferative phase of the cell cycle.

In TCGA, whole-genome sequencing results, 57 mutations in the *GMNN* gene were identified in 13 cancer types. The mutations gave rise to 51 variants of geminin, many of which were discretely distributed. Most of the 57 mutations were located in the exon region, while one was located in the 5′ untranslated region (UTR) (T > G at the chromosome position 24777229) and eight were present in the 3′ UTR (Figure 1B). There were only missense or synonymous mutations at the N terminus of the protein, whereas deletions, nonsense mutation, and splice site mutations were also observed at the C terminus (Figure 1C). An arginine at amino acid position 54 was more susceptible to glutamine substitution than other amino acids (Figure 1C). Thus, mutations in the *GMNN* gene are not widespread in human cancers. As cells alter the expression of replication factors to conquer replicative stress in the early stages of tumorigenesis, mutations in replication factors are rare.

### Depletion of Geminin Selectively Induces DNA Re-Replication in Gastric Cancer Cells

**FIGURE 2:**
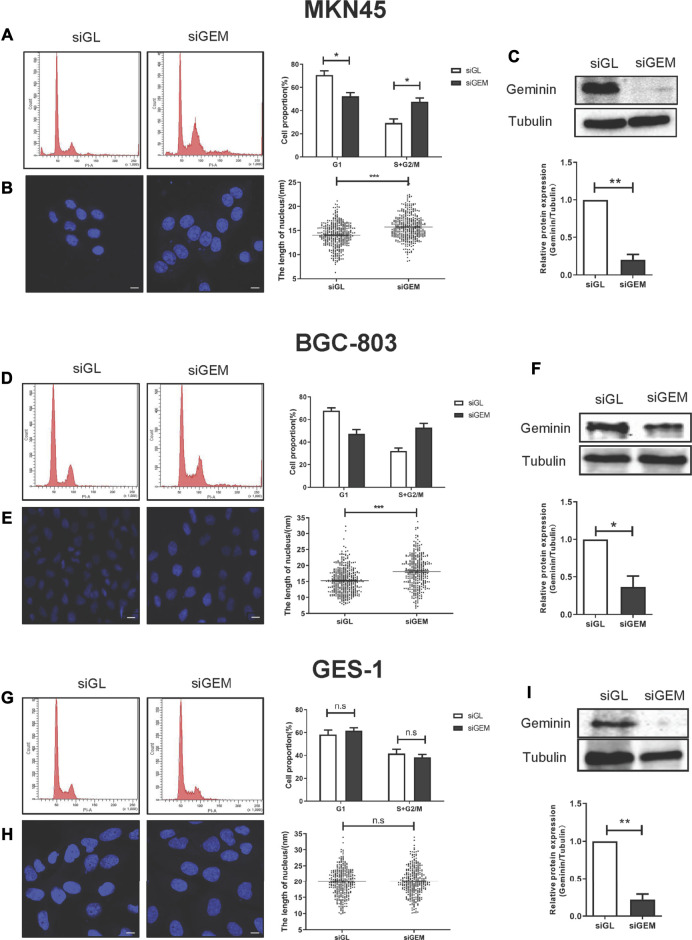
Depletion of geminin induces DNA re-replication in gastric cancer cells but not in normal gastric epithelial cells. Two gastric cancer cells (MKN45 and BGC-803) and normal epithelial cells (GES-1) were transfected with siGL or siGEM. After 48-h posttransfection, the cells were harvested and stained with PI to quantify DNA content by FACS analysis **(A, D, G)** or with DAPI to visualize nuclei by LSM **(B, E, H)**. Geminin and tubulin were detected by Western blotting **(C, F, I)**. Densitometory of three independent experiments was performed, normalized to tubulin, and expressed as a ratio of siGL, respectively, mean ± SD, *p < 0.05, **p < 0.01, ***p < 0.001 (T test).

SiRNAs targeting *GMNN* (siGEM) were shown to induce DNA re-replication in colorectal carcinoma, head and neck squamous cell carcinomas (HNSCCs), and breast cancer (Zhu et al., 2004; Zhu and Depamphilis, 2009). However, this effect varied according to cell type: immortalized cells derived from normal tissues and some cancer cell lines (e.g., cervix adenocarcinoma cells HeLa, skin melanoma cells A375 and WM-266-4) were resistant to DNA re-replication induced by *GMNN* knockdown (Zhu and Depamphilis, 2009). We examined the effect of geminin depletion in gastric cancer using three gastric cell lines (MKN45, BGC-803, and GES-1 cells). Two days after transfection with siGEM, the proportion of gastric cancer cells MKN45 and BGC-803 in the S + G2/M phase was increased compared to the cells transfected with a control siRNA (siGL) (Figures 2A,D), and it was accompanied by an increase in the number of giant nuclei observed by laser scanning confocal microscopy (Figures 2B,E) and downregulation of geminin expression (Figures 2C,F). Depletion of geminin had no effect on the viability and proliferation of MKN45 and BGC-803 cells (Supplementary Figure 1).

We examined whether depletion of geminin had the same effect on GES-1 cells, a normal gastric epithelial cell line. As expected, siGEM reduced geminin expression (Figure 2I) but no changes were observed in the proportion of cells in the S + G2/M phase (Figure 2G) or in the fraction of cells with giant nuclei (Figure 2H). Thus, depletion of geminin induces DNA re-replication in gastric cancer cells but not in normal gastric epithelial cells.

### LPA Selectively Triggers the Upregulation of Geminin in Gastric Cancer Cells

**FIGURE 3:**
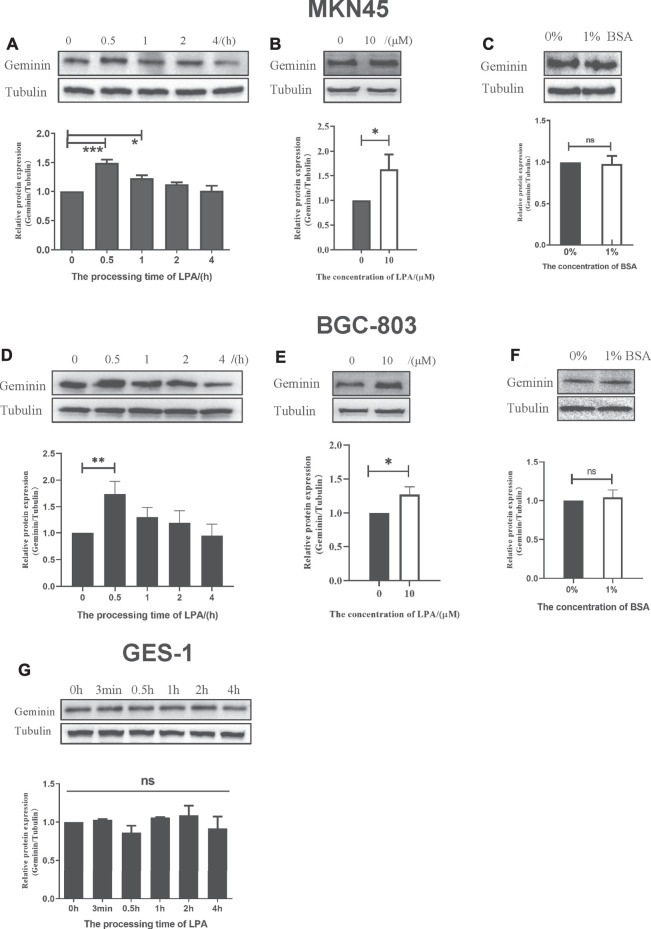
LPA selectively triggers the upregulation of geminin in gastric cancer cells. **(A, D, G)** G1/S–arrested cells were treated with LPA time gradient. **(B, E)** Protein level of geminin with or without 10 µM LPA treatment. **(C, F)** G1/S–arrested cells were under the treatment with or without 1% BSA. Densitometry of three independent experiments was performed, normalized to tubulin and expressed as a ratio of 0 h or 0 µM, respectively. Mean ± SD, *p < 0.05, **p < 0.01 (ANOVA test and Dunnett’s multiple comparisons test )

LPA is a GPCR agonist which can modulate cell proliferation (Gschwind et al., 2002), migration (Guo et al., 2015), and invasion (Gschwind et al., 2003) *via* multiple signaling pathways. Abnormalities in LPA signal transduction can lead to cancer development and metastasis (Mills and Moolenaar, 2003). To investigate the role of LPA in gastric cancer, MKN45, BGC-803, and GES-1 cells were treated with LPA, and geminin protein level was evaluated by Western blotting. 10 μM LPA treatment could induce a significant increase in the geminin expression (Figures 3B,E). LPA stimulation caused a transient upregulation of geminin in the early S phase (0–2 h) in MKN45 cells (Figure 3A) and BGC-803 cells (Figure 3D) but not GES-1 cells (Figure 3G). Prior to LPA treatment, the cells were serum starved and maintained in a quiescent state, and 1% BSA was added to the medium to preserve the biological activity of LPA. To exclude the effect of BSA on geminin expression, we evaluated the changes in the protein level following incubation for 0.5 h in the presence or absence of 1% BSA. As expected, BSA did not affect geminin protein levels in gastric cancer cells (Figures 3C,F). These results indicate that LPA specifically increases geminin protein level in gastric cancer cells.

As a DNA replication factor, geminin shuttles among the nucleus, nucleoplasm, and cytoplasm (McGarry and Kirschner, 1998). In normal human gastric tissue, geminin was localized in the cytoplasm and cytoplasmic membrane of all tissues and was detected in the nucleus only in 67% of samples *via* IHC. In contrast, geminin was detected in the nucleus of 81.8% of tumor tissue samples, with exclusive nuclear localization in 72.73% (data were obtained from HPA, Supplementary Figure 2A).

To investigate whether LPA affects cytoplasmic–nuclear trafficking of geminin in gastric cancer, we examined geminin localization at different time points after LPA treatment. In quiescent cells treated with DMSO, geminin was mainly detected in the cytoplasm, and nuclear localization increased with prolonged LPA treatment (Supplementary Figure 2B). Thus, in gastric cancer cells, LPA stimulation promotes nucleus transfer of geminin, which can be a useful diagnostic marker.

### LPA Enhances Geminin Stability Through Deubiquitinating Enzyme DUB3 in Gastric Cancer Cells

**FIGURE 4:**
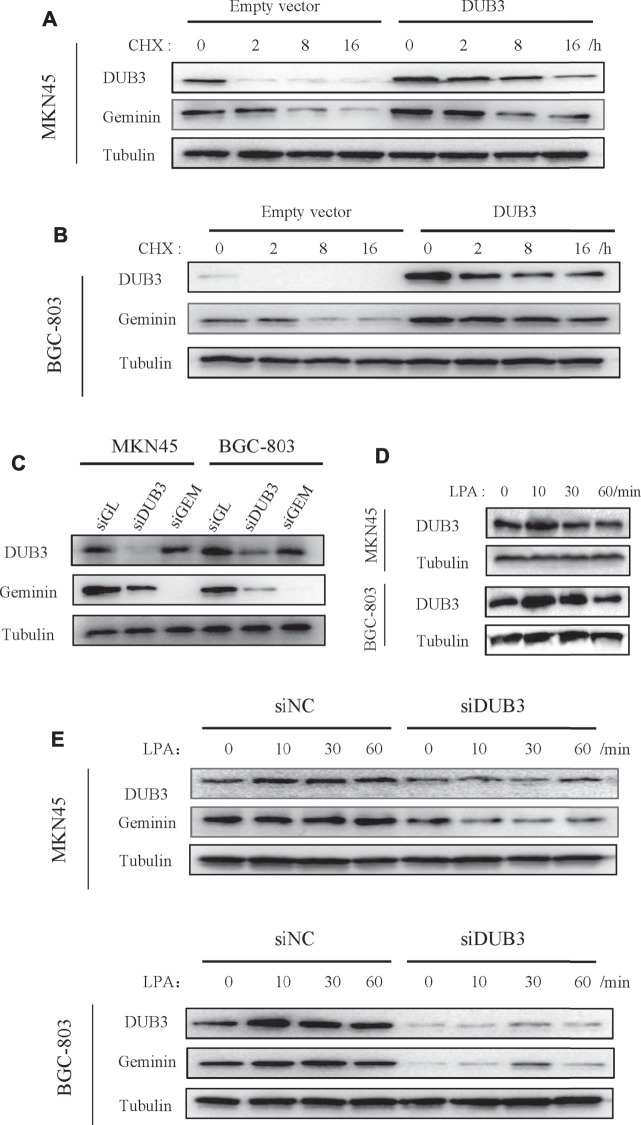
LPA potentiates geminin stability through DUB3 in gastric cancer cells. **(A, B)** Gastric cancer cells MKN45 **(A)** and BGC-803 **(B)** were transfected with the indicated vectors (empty vector; DUB3, a vector that contains *Homo sapiens* ubiquitin-specific peptidase 17-like family member 2 [USP17L2] mRNA [DUB3]), and incubated with cycloheximide (CHX, 50 µg/ml) for the indicated time points. Western blotting analysis with the indicated antibodies. **(C)** Two gastric cancer cells (MKN45 and BGC-803) were transfected with the indicated siRNA. Cell lysates were analyzed by Western blotting. **(D)** Two gastric cancer cells were treated with LPA time gradient, and cell lysates were analyzed by Western blotting. **(E)** Two gastric cancer cells (MKN45 and BGC-803) were transfected with the indicated siRNA, together with LPA time gradient treatment, and cell lysates were analyzed by Western blotting.

APC/C controls geminin degradation, while DUB3 is also known to regulate geminin protein stability (Hernández-Pérez et al., 2017). The latter was confirmed by overexpressing transfected DUB3 in gastric cancer cells, which were then treated with cycloheximide to block new protein synthesis, followed by measuring geminin protein levels. DUB3 increased geminin stability compared to that of the control empty vector (Figures 4A,B). Conversely, *DUB3* knockdown significantly decreased geminin protein levels in MKN45 and BGC-803 cells (Figure 4C).

To investigate the relationship between LPA-induced upregulation of geminin and its deubiquitination by DUB3, we assessed the expression levels of DUB3 protein following LPA stimulation by Western blotting. DUB3 was upregulated in MKN45 and BGC-803 cells, with peak expression 10 min after LPA treatment (Figure 4D); meanwhile, geminin protein levels reached a maximum within 1 h (Figure 3). Next, the changes of DUB3 and geminin protein expression after the depletion of DUB3 together with LPA stimulation were observed. Indeed, *DUB3* knockdown significantly affected geminin stability (Figure 4E). These results suggest that LPA enhances the stability of geminin in gastric cancer cells by inducing its deubiquitination through DUB3.

### LPA Stimulates EGFR Transactivation *via* an MMP-Dependent Pathway in Gastric Cancer Cells

**FIGURE 5:**
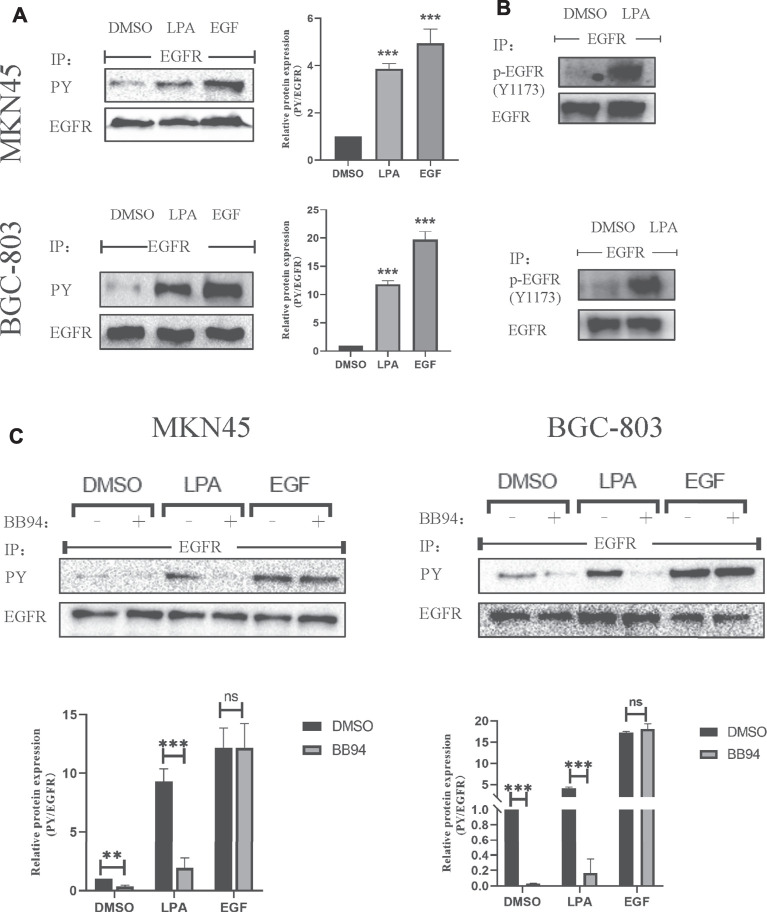
LPA stimulates EGFR transactivation in gastric cancer cells. **(A–B)** Tyrosine-phosphorylated EGFR (**(A)**, PY) and Y1173-phosphorylated EGFR **(B)** level after treated with 0.1% DMSO (vehicle), 10 µM LPA, or 10 ng/ml EGF for 3 min. **(C)** Tyrosine-phosphorylated EGFR levels after pretreated with 0.1% DMSO or 10 µM BB94 for 30 min and stimulated with 10 µM LPA. Densitometry of three independent experiments was performed normalized to tubulin and expressed as a ratio of DMSO; mean ± SD, **p < 0.01 (ANOVA test and Dunnette’s multiple comparisons test).

LPA can induce an intracellular transactivated mechanism, and it is thought to activate GPCR-regulated transmembrane MMPs at the cell surface, leading to EGFR transactivation via a classic autocrine mechanism (Daub et al., 1996; Prenzel et al., 1999). To determine whether LPA is involved in EGFR transactivation in gastric cancer, we first examined the changes in EGFR phosphotyrosine level upon LPA (10 μM) stimulation. In two of the tested gastric cancer cell lines, tyrosine phosphorylation of EGFR was increased in the presence of LPA. In contrast, the vehicle DMSO had little effect, although EGF-induced EGFR autophosphorylation was obviously enhanced (Figure 5A). Moreover, LPA treatment increased phosphorylation at Y1173, a well-known transphosphorylation site for EGFR activation (Figure 5B). These results provide evidence for the cross-talk linking between GPCR with EGFR signal pathway in gastric cancer cells.

MMPs mediate the cross-talk between GPCR and EGFR in HEK-293 cells (Prenzel et al., 1999) and HNSCCs cells (Gschwind et al., 2002). We therefore examined whether MMPs is involved in LPA-induced EGFR transactivation in MKN45 and BGC-803 cells in the absence or presence of the MMP inhibitor batimastat (BB94). BB94 (10 μM) completely blocked the EGFR transactivation induced by LPA treatment, whereas EGF-induced EGFR autophosphorylation was unaffected (Figure 5C). In addition, BB94 also affected the basic EGFR autophosphorylation in gastric cancer, perhaps by modulating the activity of basic EGFR ligand (Figure 5C). These results indicate that LPA induces EGFR transactivation in gastric cancer via an MMP-dependent mechanism.

### LPA Potentiates Geminin Stability *via* the LPAR3/MMPs/EGFR/PI3K/mTOR Signaling

**FIGURE 6:**
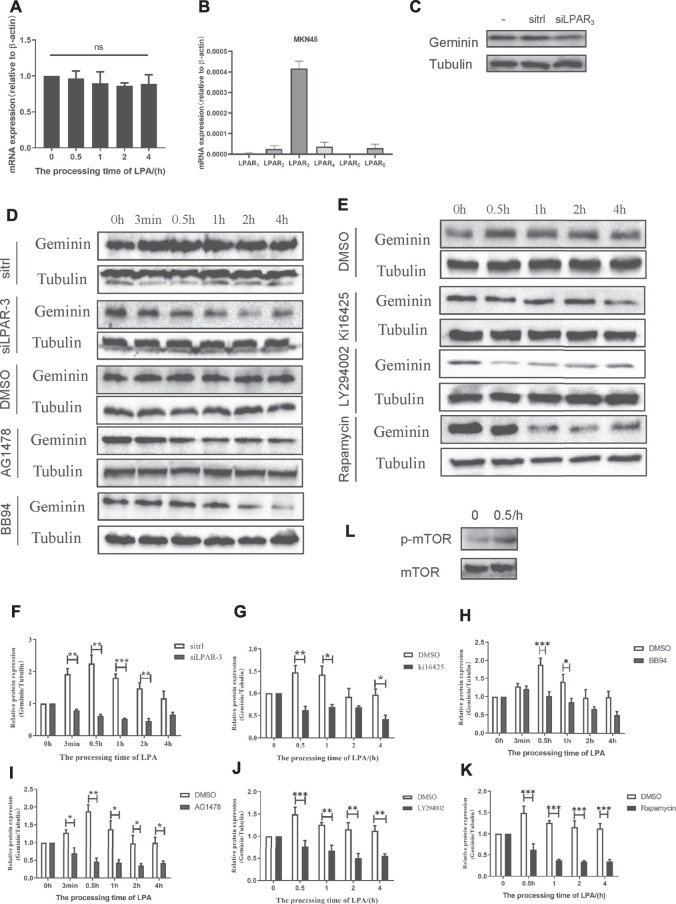
LPA potentiates geminin stability through LPAR_3_/MMP_S_/EGFR/PI3K/mTOR signaling axis in MKN45 cells. **(A)** mRNA expression of geminin under 10 µM LPA time gradient. Densitometry of three independent experiments was performed normalized to actin and expressed as ratio of 0 h; mean ± SD (ANOVA test and Dunnett’s multiple comparisons test). **(B)** mRNA expression of LPARI-6 in MKN45 cells. **(C)** Geminin protein levels after transfected with or without sitrl/siLPAR3 for 6 h, and the cells were cultured for another 48 h in the total medium until harvested. **(D–E)** Gemimin protein level after pretreated with DMSO (vehicle, 0.1%), Kil6425 (10 µM), BB94 (10 µM), AG1478 (250 nM), LY294002 (10 µM), or rapamycin (100 nM) for 30 min, and stimulated with 10 µM LPA time gradient. In siRNA-silencing experiments, cells were transfected with either siGL or siGEM for 6 h and then cultured for another 48 h. **(F–K)** Quantification of geminin protein levels in **(D)** and **(E)**. Densitometry of three independent experiments was performed normalized to tubulin and expressed as a ratio of control group; mean ± SD, *p < 0.05, **p < 0.01, ***p < 0.001 (ANOVA test and Dunnette’s multiple comparisons test). **(L)** Activation level of mTOR and p-mTOR (Ser2448) under 10 µM LPA treatment.

We showed that LPA treatment increased geminin protein levels in gastric cancer cells during the S phase. To elucidate the signaling pathway(s) involved, we examined the mRNA level of *GMNN* under the time gradient treatment of LPA by qRT-PCR. Unlike the effect on geminin protein level, *GMNN* transcript level was not altered by LPA treatment in MKN45 cells (Figure 6A) and BGC-803 cells (Supplementary Figure 3A).

Of the six LPARs, LPAR1, LPAR2, and LPAR3 are most widely studied. To identify the LPAR(s) involved in LPA-induced EGFR transactivation in gastric cancer, we evaluated the mRNA levels of various LPARs by qRT-PCR and found that LPAR3 was more highly expressed than the others (Figure 6B; Supplementary Figure 3B). Knockdown of *LPAR3* reduced the protein level of geminin that was increased by LPA treatment (Figures 6D,F), whereas no change in geminin expression was observed in gastric cancer cells that were transfected with a control siRNA (sitrl) or siLPAR3 in the absence of LPA (Figure 6C). To confirm the role of LPAR3 in LPA-meditated geminin upregulation, we evaluated geminin protein levels in gastric cancer cells treated with the LPAR1/3 inhibitor Ki16425 by Western blotting. Ki16425 (10 μM) completely abrogated the increase in geminin protein expression induced by LPA in MKN45 cells (Figures 6E,G) and BGC-803 cells (Supplementary Figures 3C,D), whereas the vehicle DMSO (0.1%) had no effect, indicating that LPA regulates geminin stability through LPAR3.

To investigate the relationship between the stabilization of geminin and EGFR transactivation induced by LPA, we measured geminin protein levels in cells treated with DMSO, the MMP inhibitor BB94 (10 μM), or the EGFR inhibitor AG1478 (250 nM) by Western blotting. Gastric cancer cells were synchronized with serum-free medium for 24 h and then pretreated with a vehicle or inhibitors for 30 min before treatment with LPA (10 μM) for different times. Inhibitors treatment completely abrogated the upregulation of geminin protein under LPA stimulation in MKN45 cells (Figures 6D,H,I) and BGC-803 cells (Supplementary Figures 3C,E,F), while DMSO had no effect, indicating that LPA regulates geminin stability via EGFR transactivation signal pathway.

GPCR-induced EGFR transactivation is known to activate the Ras-MAPK pathway in some cell lines, including GT1-7, COS-7, and HEK-293 cells (Shah et al., 2003). However, inhibiting MAPK signaling using MAPK kinase (MEK) or extracellular signal-regulated kinase (ERK) inhibitors did not attenuate LPA-induced geminin upregulation (data not shown). We investigated the downstream factor(s) of EGFR involved in LPA-mediated geminin stabilization, and found that in both MKN45 and BGC-803 cells LPA treatment increased Phospho-mTOR (Ser2448) level (Figure 6L; Supplementary Figure 3I). Moreover, the PI3K inhibitor LY294002 (10 μM) and mTOR inhibitor Rapamycin (100 nM) notably eliminated the upregulation of geminin protein stimulated with LPA in MKN45 cells (Figures 6E,J,K) and BGC-803 cells (Supplementary Figures 3C,G,H), whereas DMSO had no effect. Thus, LPA enhances geminin stability *via* the LPAR3/MMPs/EGFR/PI3K/mTOR signaling axis in gastric cancer.

### LPA Mediates S-Phase Cell-Cycle Progression *via* the LPAR_3_/MMPs/EGFR/PI3K/mTOR Signaling

**FIGURE 7:**
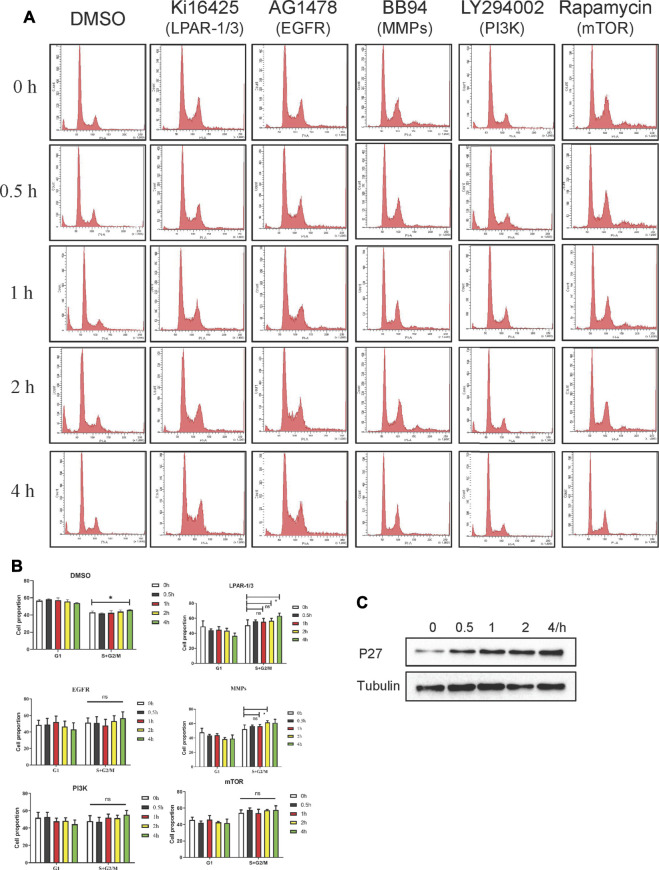
LPA induces S phase cell-cycle progression through LPAR_3_/MMP_S_/EGFR/PI3K/mTOR signaling axis in MKN45 cells. **(A)** Quiescent cells were pretreated with DMSO (vehicle 0.1%), Kil6425 (10 µM), BB94 (10 µM), AG1478 (250 nM), LY294002 (10 µM), or rapamycin (100 nM) for 30 min and stimulated with 10 µM LPA up to 4 h. After the treatment, the cells were harvested and stained with PI to analyze the quality of DNA content by FACS analysis. **(B)** Qualification of DNA content in **(A)**, mean ± SD, *n* = 3. *p < 0.05, ANOVA test and Dunette’s multiple comparisons test were used for data analysis. **(C)** Expression of p27 after LPA time grade stimulation.

Cell cycle–related proteins other than geminin such as cell division cycle (Cdc)6, Cdt1, minichromosome maintenance complex component (Mcm)2, Mcm7, and Mcm10 did not respond to LPA stimulation in preliminary experiment (data not shown); however, the expression of the cell cycle–related protein P27 was upregulated (Figure 7C; Supplementary Figure 4C). Given our observation, LPA stabilized geminin via the LPAR3/MMPs/EGFR/PI3K/mTOR signaling axis in gastric cancer cells, we examined whether this pathway modulated cell-cycle progression. Gastric cancer cells were synchronized with serum-free medium for 24 h, and G1/S–arrested cells were pretreated with vehicle or inhibitor as described above for 30 min and then stimulated with LPA (10 μM) in a time gradient. The cells harvested and stained with PI and DNA content were quantified by flow cytometry. In both MKN45 and BGC-803 cells, inhibitor treatment did not alter the S and G2/M phase fractions compared to the cells treated with DMSO (Figures 7A,B; Supplementary Figures 4A,B). Thus, LPA promotes S-phase cell-cycle progression in gastric cancer cells via the LPAR3/MMPs/EGFR/PI3K/mTOR signaling axis.

### MMPs-Dependent Tansactivation of EGFR is Critical for LPA-Induced Efficient DNA Synthesis and Cell Proliferation in Gastric Cancer Cells

**FIGURE 8:**
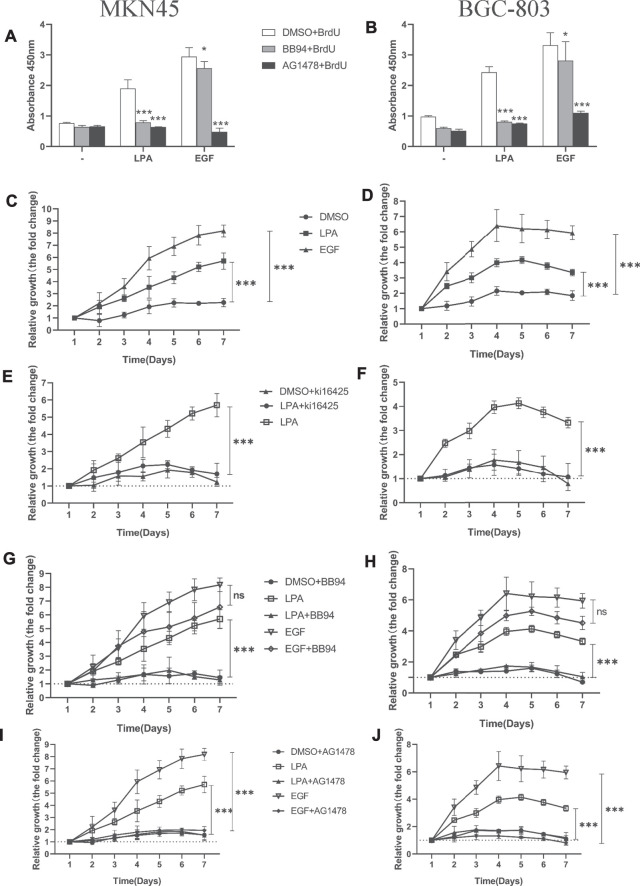
Kil6425, BB94, and AG1478 inhibit LPA-induced efficient DNA synthesis and cell proliferation in gastric cancer cells. **(A–B)** LPA-induced efficient DNA synthesis after pretreated with inhibitors for 30 min and grown in the ligands (0.1% DMSO, 10 µM LPA, or 10 ng/ml EGF) for 4 days. **(C–D)** Cell proliferation of gastric cancer cells with ligands. **(E–J)** Cell proliferation of gastric cancer cells after pretreated with inhibitors for 30 min and grown in the ligand. In qualifications, the cell growth were calculated using GraphPad Prism v8.0 software at each time point, and the fold is compared relative to the level at 1 day, respectively, mean ± SD, *n*=3, *p < 0.05, ***p < 0.001 (ANOVA test and Dunnette’s multiple comparisons test).

To quantify the efficiency of DNA synthesis in response to LPA stimulation, we evaluated the ratio of DNA synthesis by measuring BrdU incorporation using ELISA assay. In both MKN45 and BGC-803 cells, BB94 and AG1478 notably abrogated DNA synthesis induced by LPA, respectively (Figures 8A,B). Furthermore, AG1478 notably eliminated DNA synthesis upon exogenous EGF stimulation (Figures 8A,B). It is surprising to observe that BB94 also reduced DNA synthesis induced by exogenous EGF in gastric cancer cells. This suggested that exogenous EGF stimulation may result in enhancing the shedding of endogenous EGFR ligands in gastric cancer as observed in HNSCCs (Gschwind et al., 2002; O-charoenrat et al., 2001). Taken together, these data strongly indicate that LPA-induced efficient DNA synthesis is dependent on metalloprotease function in gastric cancer.

An increase in cell proliferation is another hallmark of tumorigenesis. We investigated whether MMPs or EGFR inhibition influences LPA-induced efficient cell proliferation in gastric cancer cells with the CCK-8 assay. Treatment with LPA or EGF enhanced efficient cell proliferation in MKN45 (Figure 8C) and BGC-803 cells (Figure 8D); this was blocked by the LPAR1/3 inhibitor Ki16425 (Figures 8E,F) and by BB94 (Figures 8G,H) and AG1478 (Figures 8I,J). Notably, BB94 had no effect in EGF-induced cell proliferation (Figures 8G,H). These results demonstrate that LPAR3, MMPs, and EGFR activities are required for LPA-induced efficient DNA synthesis and cell proliferation in gastric cancer.

## Discussion

**FIGURE 9:**
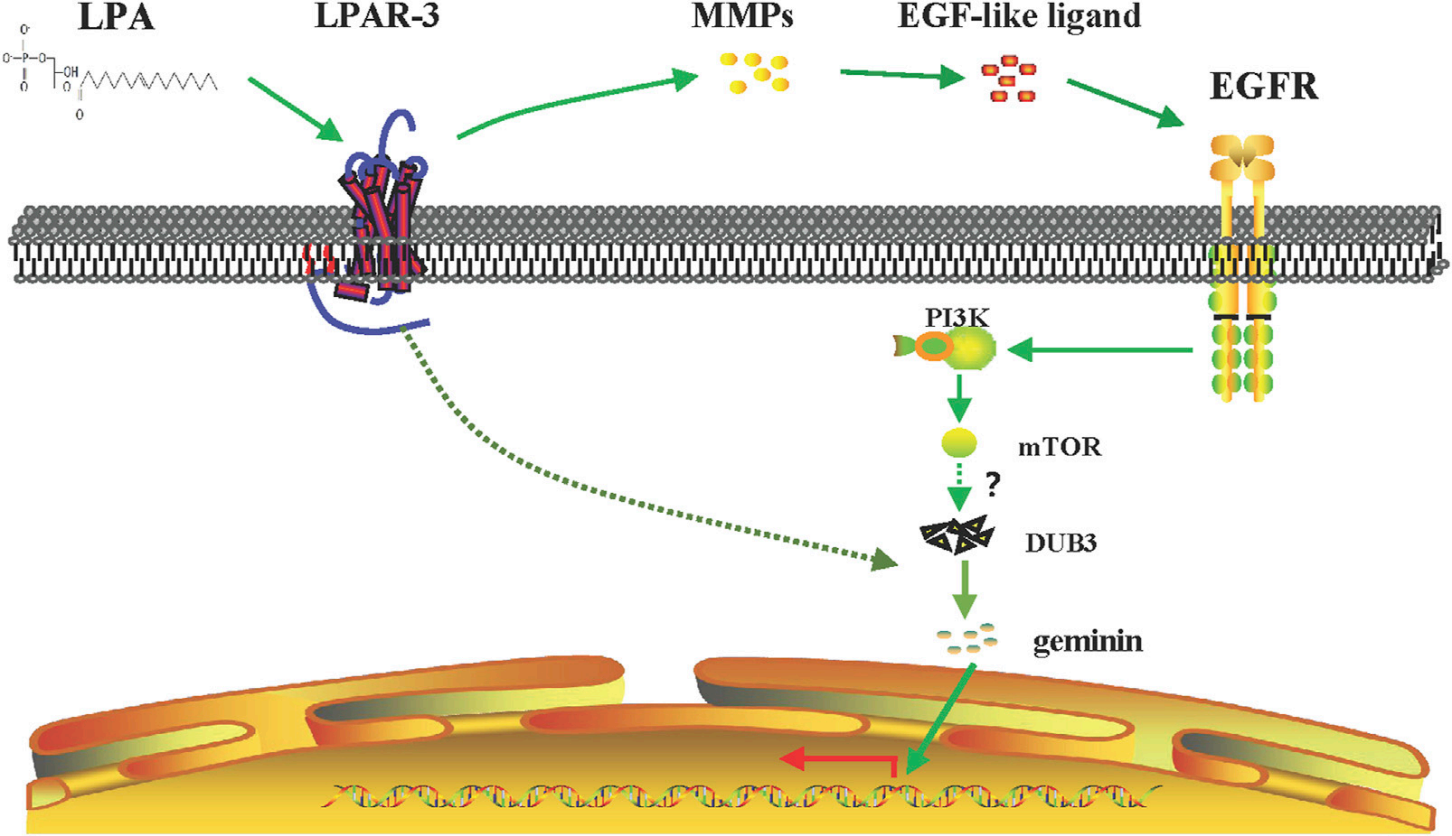
Signaling pathway of LPA-mediated DNA replication initiation. LPA work through LPAR_3_ to transactive EGFR by MMPs and to increase the expression of geminin protein level in the S phase through PI3K/mTOR signaling pathway. Meanwhile, LPA stimulation induces upregulation of DUB3 in a short time, inhibiting the ubiquitination degradation of geminin protein and enhancing its stability, and then positively regulating the DNA replication initiation of gastric cancer cells.

In this study, we showed that geminin mutations are not widespread in human cancers, consistent with its function as a regulatory protein. Depletion of geminin selectively induced DNA re-replication in gastric cancer cells but not in normal gastric epithelial cells, whereas LPA treatment induced the upregulation of geminin protein in the S phase in gastric cancer cells. Notably, LPA stimulated EGFR transactivation via an MMPs-dependent mechanism in gastric cancer, which was partly responsible for enhancing geminin stability in the S phase and promoting DNA replication. On the other hand, LPA also induced the rapid upregulation of DUB3; thereby, indirectly stabilizing geminin by preventing its degradation. These data indicate that the cross-talk between LPA and EGFR signaling regulates DNA replication by stabilizing geminin level in the S phase to promote gastric cancer progression (Figure 9).

The survival of tumor cells depends on their interaction with components of the TME including ECM, surrounding vasculature, other non-malignant cells, and signaling molecules (Quail and Joyce, 2013). Perturbation of the TME promotes cancer cell transformation and invasion of other tissues, facilitating cancer progression (Roy and Walsh, 2014). Many cancers are characterized by overexpression of LPA, which is also present in the ECM (Nouh et al., 2009). There is increasing evidence that LPA is an important factor in the TME which promotes tumor growth, migration, invasion, metastasis, and angiogenesis. LPA was found to stimulate cell migration and invasion mainly via LPAR1-3 (Shida et al., 2008; Ren et al., 2019); however, the role of LPA/LPARs in DNA replication has not been reported. Here, we show for the first time that LPA promotes DNA replication by up-regulating geminin protein in gastric cancer cells. As an oncogene, geminin is overexpressed in tumors, which is linked to the prognosis of colon, rectal, and breast cancers (Montanari et al., 2005; Hernández-Pérez et al., 2017; Zhang et al., 2017). The expression of geminin is cell cycle–dependent, and the protein is synthesized during the S phase with a half-life of 3–4 h (Kulartz et al., 2003). We demonstrated that LPA treatment increased geminin expression in the S phase in gastric cancer cells. This effect depends on cellular context, as LPA was shown to induce the upregulation of geminin in gastric and ovarian cancer cells but not in breast cancer cell line MCF-7 that frequently overexpress the protein (data not shown), although the reasons for this difference are not clear. Deregulation of geminin has been reported in different human cancers and is associated with DNA replication and metastasis (Hernández-Pérez et al., 2017; Zhang et al., 2017). Our data establish a novel link between geminin-regulated DNA replication and tumor progression; an elevated level of LPA in the TME of gastric cancer cells enhanced the stability of geminin in the S phase, which promoted rapid DNA replication and tumor progression.

Identifying patient subpopulations that would benefit from a personalizing targeted therapeutic regimen is important for improving clinical outcomes. Mutations in specific genes can alter the proliferative potential of tumor cells. Although we found that mutations or deletions of the *GMNN* gene were not prevalent in human cancers, the R54Q mutation in geminin protein was detected at a high frequency, and other potentially significant sites that may be phosphorylated under different circumstances are Thr25, S32, S60 (Tsunematsu et al., 2013) S45, and S49 (Kulartz et al., 2003), although the physiologic relevance of these modifications remains to be determined.

Mutations in replication factors are rare in cancer; tumor cells have adopted other strategies to alter the levels of replication factors to induce replicative stress. Geminin is downregulated in G0/G1 phases by APC/C E3 ubiquitin ligase (McGarry and Kirschner, 1998) in association with polycomb group complex 1 and the RDCOX complex (containing Scmh1Hoxb4/Hoxa9), which are associated with E3 ubiquitin ligase core complex Roc1/Ddb1/Cul4a (Ohno et al., 2013). Recent study certifies that DUB3 and USP7 were shown to control geminin protein stability in breast cancer (Hernández-Pérez et al., 2017); our results demonstrate that DUB3 also regulates geminin protein stability in gastric cancer.

Activation or inhibition of protein tyrosine phosphorylation signaling networks can lead to consequent changes in gene transcription and translation. We identified a novel signaling axis that modulates geminin expression through LPA-induced EGFR (Y1173) transactivation in gastric cancer cells. EGFR initiates a signaling cascade that leads to DNA synthesis and cell proliferation; and EGFR and PI3K initiate malignant transformation of cancer cells by activating other pathways such as Myc and Rb-E2F, which in turn leads to the formation of cyclinD/Cdk4 and cyclinE/Cdk2 complexes (Read et al., 2009). In the early S phase, Cdk2 phosphorylates c-Myc and promotes its association with the promoter of miR-571, thereby inhibiting its expression. miR-571 targets *GMNN* mRNA, and its downregulation is associated with the accumulation of geminin protein (Zhang et al., 2019). This may elucidate one possible signaling event downstream of LPA-induced geminin upregulation. Aurora-A phosphorylates geminin on Thr25 to protect it from APC/C-dependent proteolysis during the early M phase, and the binding of geminin to Cdt1 protects the latter from ubiquitylation and proteolysis (Tsunematsu et al., 2013). We investigated whether LPA stimulation leads to geminin phosphorylation, but were unable to identify the phosphorylation site by mass spectrometry analysis (data not shown). Further studies are needed to identify the kinase acting downstream of EGFR that phosphorylates and thereby stabilizes geminin.

GPCR and EGFR are two pivotal families of drug targets, given that GPCR-induced transactivation of EGFR has been linked to cancer development (Köse, 2017), through the activation of MAPK signaling, stimulation of cell migration, and regulation of cell-cycle progression. We provided evidence that LPA-induced EGFR transactivation in gastric cancer cells promoted efficient DNA synthesis, cell-cycle progression through the S phase, and cell proliferation. Based on these findings, disrupting the cross-talk between the two receptors might be a potential therapeutic strategy for the treatment of gastric cancer.

## Data Availability

The raw data supporting the conclusions of this article will be made available by the authors, without undue reservation.
