# Supplementary figures and images for: Lysophosphatidic Acid–Induced EGFR Transactivation Promotes Gastric Cancer Cell DNA Replication by Stabilizing Geminin in the S Phase
Source: Front Pharmacol. 2021 Sep 29;12:706240. doi: 10.3389/fphar.2021.706240 (PMC8511314; doi:10.3389/fphar.2021.706240)

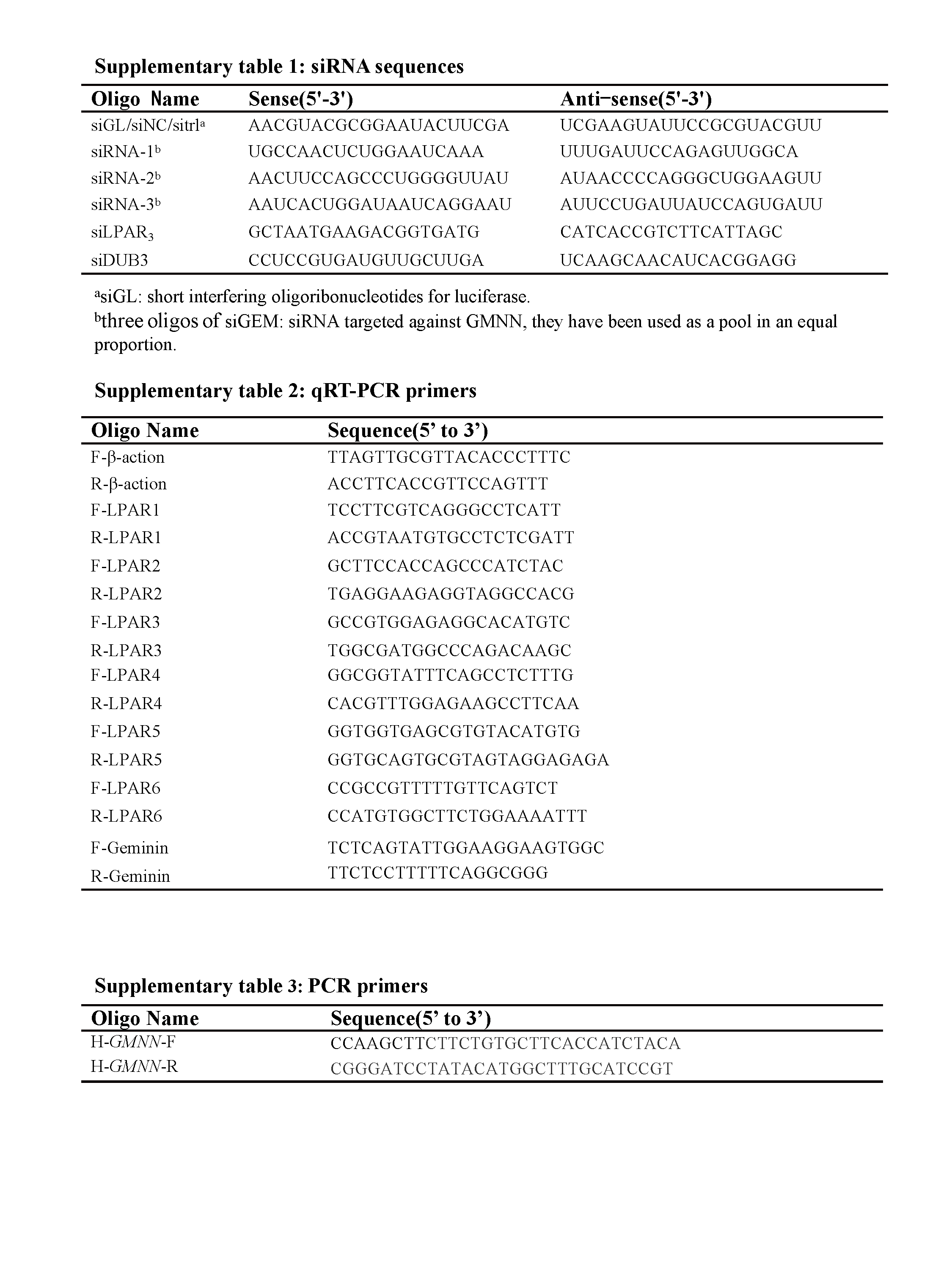

Supplement: Supplementary file 1 [file Image6.TIF]

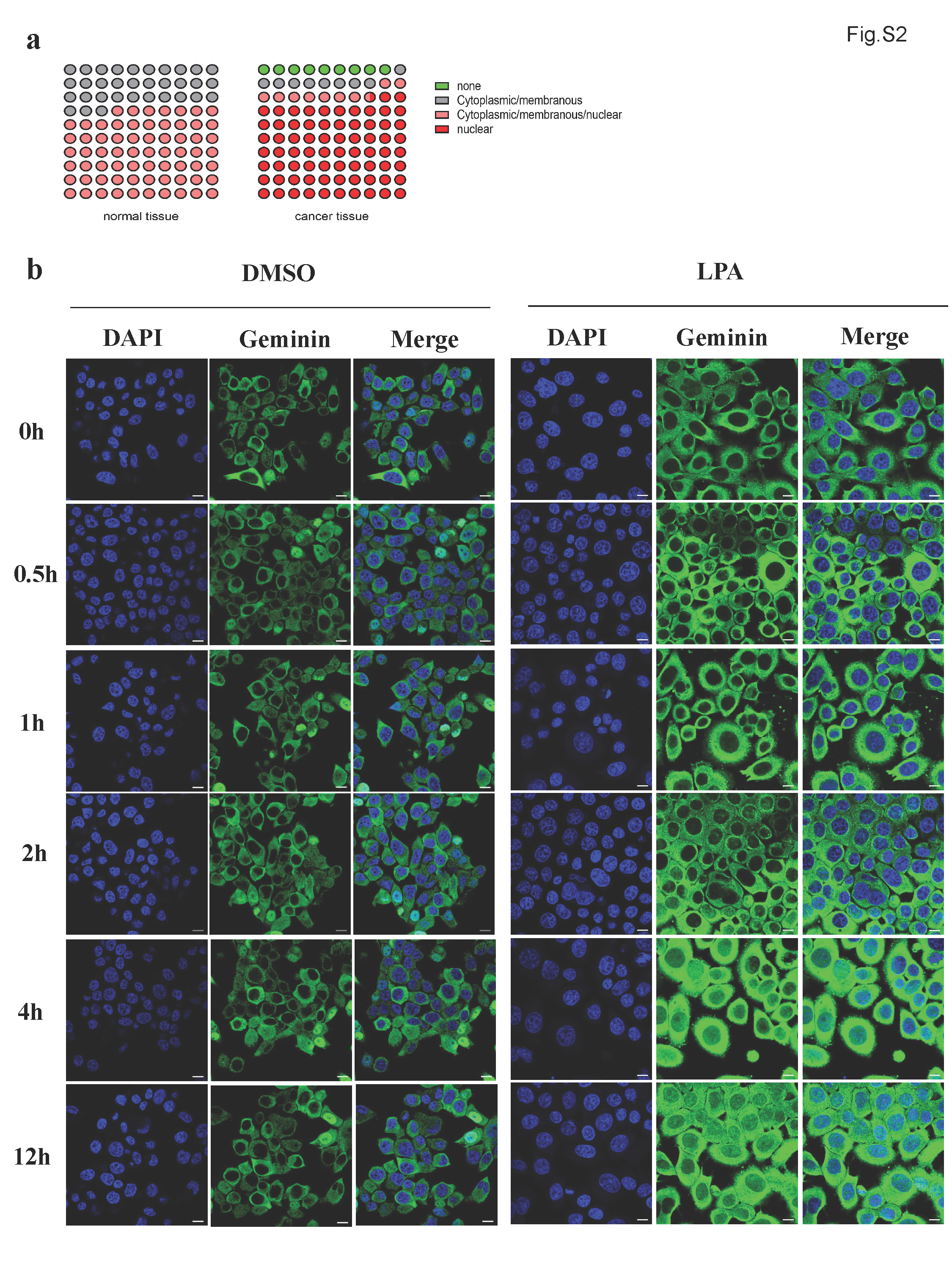

Supplement: Supplementary file 2 [file Image3.TIF]

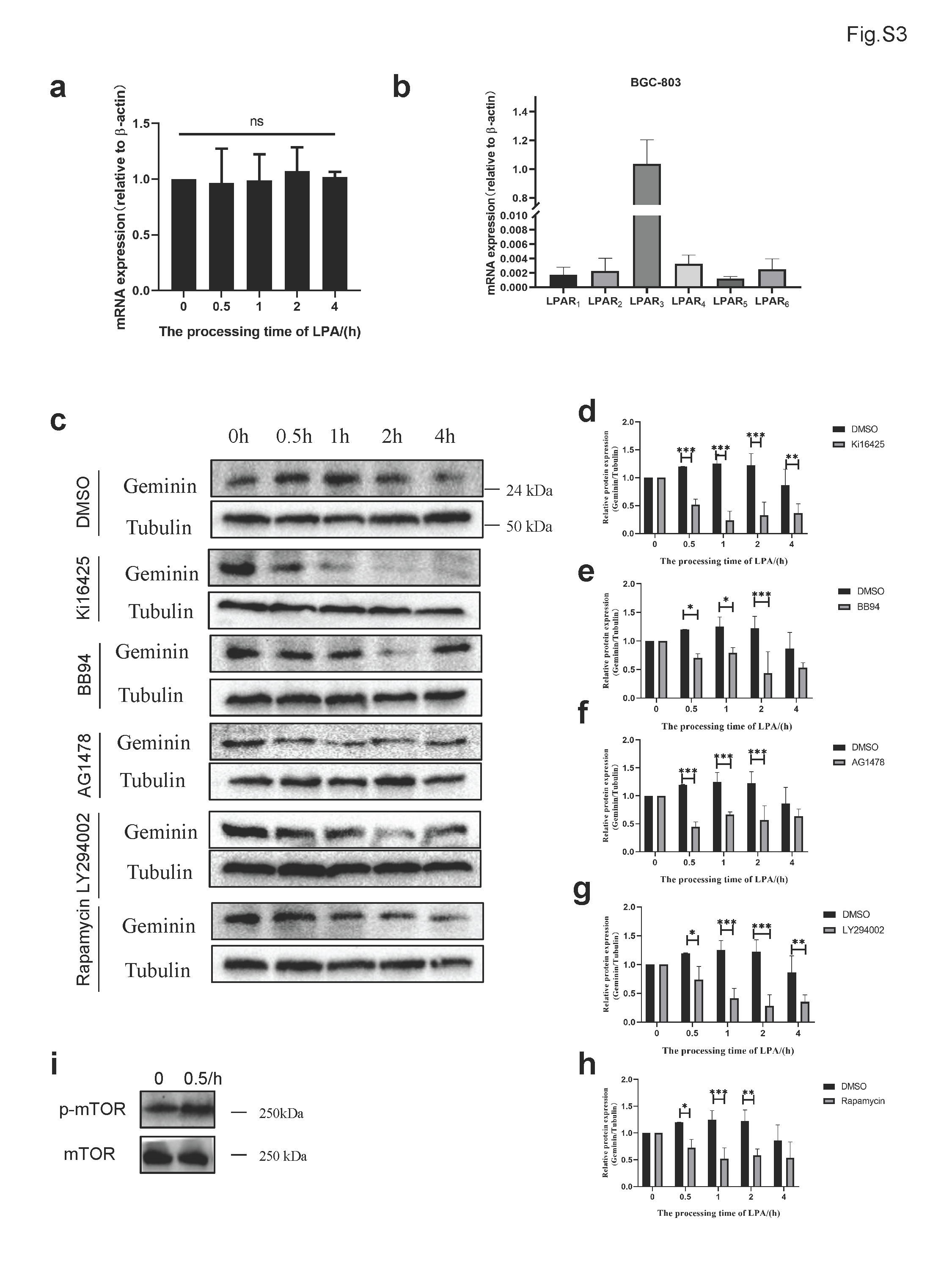

Supplement: Supplementary file 3 [file Image4.TIF]

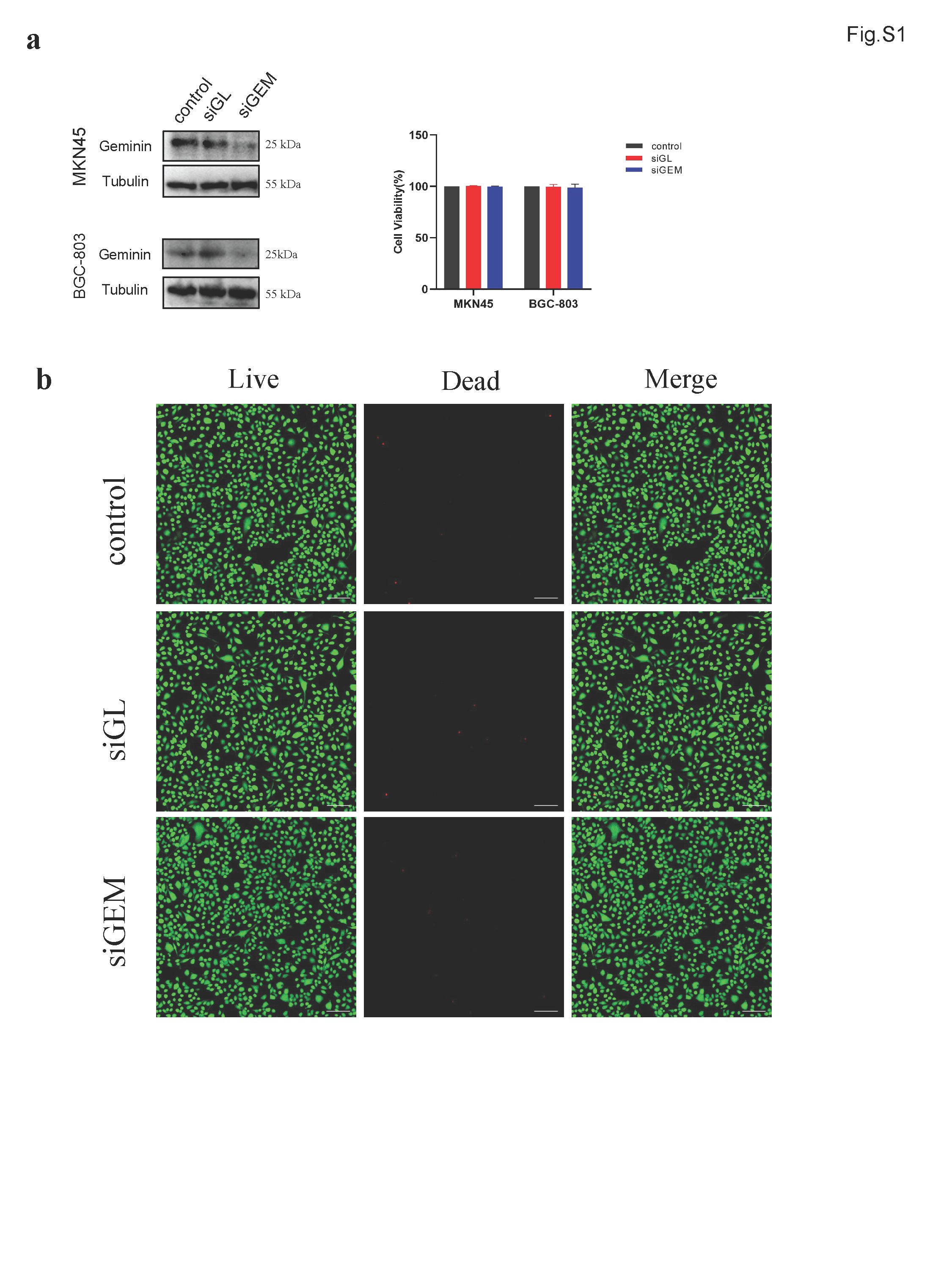

Supplement: Supplementary file 4 [file Image2.TIF]

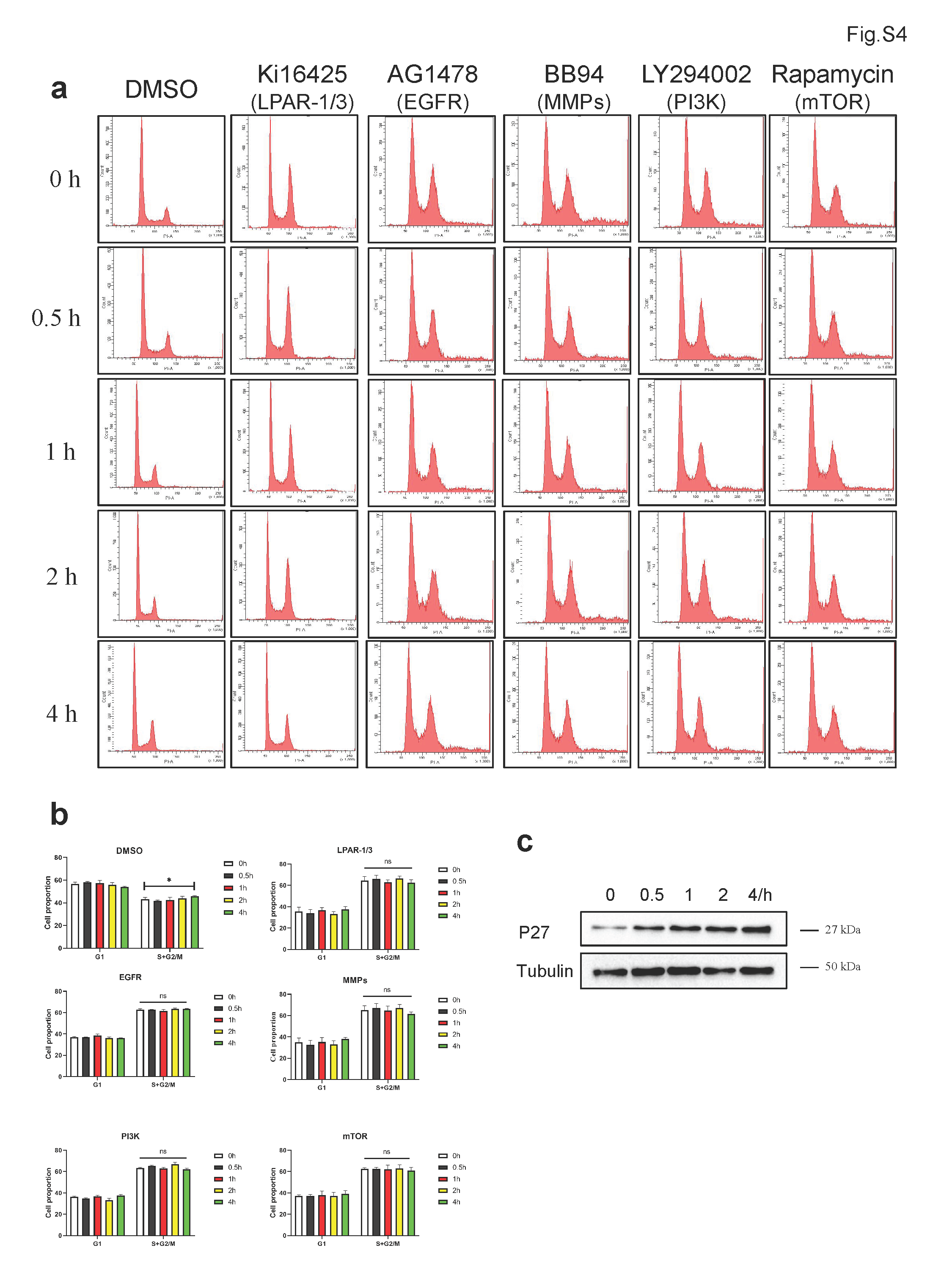

Supplement: Supplementary file 6 [file Image5.TIF]
